# A socio-constructivist framework for tactical development in team sports: fostering critical thinking through collaborative learning

**DOI:** 10.3389/fpsyg.2025.1610750

**Published:** 2025-06-13

**Authors:** Samuel Jose Octavio Gaviria Alzate, Wilder Valencia Sánchez, Elkin Arias Arias

**Affiliations:** ^1^Faculty of Education and Social Science, Technological Institution of Antioquia, Medellín, Colombia; ^2^Instituto Universitario de Educación Física y Deporte, Universidad de Antioquia, Medellín, Colombia

**Keywords:** socio-constructivist pedagogy, tactical decision-making, critical thinking in sport, collaborative learning, game-based coaching, theoretical framework, team sports

## Abstract

In team sports, tactical success depends not only on players' individual skills but fundamentally on their ability to engage in meaningful social interactions and shared understanding. This study introduces the Tactical Program based on Critical Thinking (TPCT), a pedagogical framework grounded in socio-constructivist theory, aiming to foster the development of higher-order thinking skills essential for tactical decision-making. TPCT emphasizes processes such as interpretation, analysis, inference, evaluation, explanation, and self-regulation through guided reflection and collective dialogue. Rather than imposing predefined tactical solutions, the program promotes collaborative construction of strategic responses based on reflective discussion and mutual support among teammates. Coaches using the TPCT are provided with structured guidelines to design training sessions that prioritize communication, reasoning, and joint problem-solving. By fostering an environment where knowledge is co-constructed, the TPCT contributes to more autonomous, context-sensitive, and socially informed tactical behavior. This approach offers promising applications across various team sports, reinforcing the role of critical thinking in enhancing team coordination and intelligent gameplay.

## 1 Introduction

The intrinsic relationship between cognitive processes and sports performance is well documented, particularly in relation to interpreting dynamic scenarios and selecting optimal responses during play (Gil-Arias et al., 2025; Harvey et al., 2020). Consequently, training methodologies must emphasize problem-solving and decision-making skills under pressure (Gréhaigne et al., 2005), fostering adaptability in complex and unpredictable game contexts (Hodges et al., 2021; Gaviria Alzate et al., 2024a).

Tactical performance in team sports extends beyond technical execution, relying on cognitive processes essential for making timely and effective decisions (Hallé Petiot et al., 2021). While traditional models simulate game-like situations to promote adaptability (Cesana et al., 2023), the inherent variability of collective actions limits their ability to replicate all possible game scenarios (Ashford et al., 2021). Thus, tactical competence is often cultivated both through structured drills and authentic, chaotic game contexts, with coaches shaping practice based on their tactical models (Cushion et al., 2012; Pereira et al., 2024).

A thorough understanding of the cognitive mechanisms behind effective decision-making is critical to optimize performance (Caurel and Sánchez, 2019). High-level athletes consistently demonstrate refined strategies for generating and executing tactical actions, which depend on adaptability and strategic awareness. Critical thinking, widely acknowledged as central to decision-making (Gréhaigne et al., 1999), is defined in sport as “reflective thinking used to make reasonable and defensible decisions in movement tasks” (McBride, 1992). Enhancing this cognitive skill has direct implications for tactical ability (Gréhaigne et al., 2001), yet much research still focuses on surface-level cognitive demands. As tactical efficiency requires ongoing adaptation and reflective analysis, critical thinking becomes a cornerstone for intelligent gameplay (Gaviria Alzate et al., 2024a; McBride, 1992; Rico-González et al., 2022), often neglecting the deeper processes involved in problem-solving (Rico-González et al., 2022; Silva et al., 2020).

Critical thinking enables structured reasoning, argument analysis, and strategic deduction (Gaviria Alzate et al., 2024a), and can be developed through targeted training (Lai, 2011). Evidence suggests that deliberate instruction in critical thinking leads to improved cognitive efficiency and decision quality in sport contexts (Lodewyk, 2009). Socio-constructivist models support this view, advocating for learner-centred environments where athletes actively engage with tactical problems and explore solutions collaboratively (Gaviria Alzate et al., 2024a). These approaches align with pedagogies that foster strategic planning, causal reasoning, and metacognition (McBride and Cleland, 1998), while empirical studies validate the efficacy of structured cognitive training on performance outcomes (Mahanal et al., 2019; Sari et al., 2021).

Embedding critical thinking within training routines has shown benefits in tactical awareness and real-time problem solving (Gaviria Alzate et al., 2024a; Dwyer et al., 2014). Encouraging players to critically assess strategies and performance nurtures a more informed and autonomous mindset, facilitated through feedback mechanisms and active learning (Cosgrove, 2011).

Despite increasing theoretical support, many coaches face difficulties implementing these approaches due to a lack of practical guidance (Gaviria Alzate et al., 2024a). Much of the current literature lacks accessible, applicable models for coaches outside academic settings (Gaviria Alzate et al., 2024b). Addressing this gap, the present framework highlights the transformative role of critical thinking in tactical training, providing a clear, structured methodology grounded in theory and practice (Gaviria Alzate et al., 2024a,b). It comprises:

a) A critical review of tactical development through Game-Based Approaches (GBA)
b) A practical guide for applying critical thinking in training sessions.
c) A translation of socio-constructivist principles into actionable coaching behaviors
d) A structured program of activities for developing tactical decision-making.

This approach promotes flexibility, reflective learning, and robust decision-making capacities aligned with the unpredictable demands of team sports, offering a practical solution for coaches at all levels.

### 1.1 Game-based approach (GBA) as an integrated strategy in the teaching of team sports

Team sports are inherently unpredictable, requiring players to continuously adapt tactically (Rasmussen et al., 2022; Sierra-Ríos et al., 2020). Success in these dynamic settings depends on solving problems within complex and evolving game contexts, Sierra-Ríos et al. (2020) found that TGfU interventions enhance tactical flexibility under variable game conditions. However, these findings are largely limited to youth contexts, and may require further validation in elite settings, a view aligned with Piaget's notion that learning stems from experience and adaptation (Piaget, 1952). Learning in sport is shaped by environmental and social interactions, grounded in situated and collaborative experiences (Darnis and Lafont, 2015; Godbout and Gréhaigne, 2021). In TPCT, tactical learning reflects Piagetian cycles of assimilation and accommodation, where players restructure prior knowledge to integrate novel experiences (Piaget, 1952; Hickey, 1997).

Cognition, perception, and action function as an interconnected system, supporting both technical skills and tactical understanding (Dervent et al., 2022). Thus, learning is not passive but actively driven by meaningful engagement within rich, responsive environments (Darnis and Lafont, 2015; Dervent et al., 2022).

Game-Based Approaches (GBAs) mark a pedagogical shift, combining game-like contexts with structured reflection to deepen tactical insight. Emerging in France and Germany during the 1970s and gaining traction through Bunker and Thorpe's Teaching Games for Understanding (TGfU) in the 1980s, GBAs replace instruction-led methods with facilitative coaching that encourages reflective practice and player autonomy (Ginciene et al., 2023; Harvey et al., 2018; Martínez-Santos et al., 2020). TGfU contrasts with instruction-led approaches by transferring agency to learners through structured questioning and contextualized learning, thereby enhancing autonomy (Ginciene et al., 2023; Martínez-Santos et al., 2020; Harvey and Light, 2015).

Central to GBA is understanding the internal logic of games through tactical principles that guide decision-making (Godbout and Gréhaigne, 2021). This learner-centered approach fosters intrinsic motivation, adaptability, and engagement (García-Ceberino et al., 2019; Gouveia et al., 2019), promoting improved decisions in unpredictable, high-pressure situations.

A cornerstone of GBA is the use of small-sided games (SSGs), which simultaneously develop technical skills, tactical awareness, and physical capacity (Davids et al., 2013; Dudley et al., 2024; Rodrigues et al., 2022). Their adaptable design allows manipulation of constraints to reinforce tactical principles and replicate the complexities of full games (Dervent et al., 2022; Dudley et al., 2024).

Designing effective SSGs relies on representation, which preserves the game's essence while simplifying it for learning, and exaggeration, which accentuates key tactical elements like transitions or defensive structures (Dervent et al., 2022; Dudley et al., 2024). Simple adjustments in player numbers or scoring rules can enhance individual decisions and team dynamics (Davids et al., 2013; Dudley et al., 2024).

Another essential feature is the use of structured questioning. This stimulates reflection and problem-solving by prompting players to verbalize decisions (Sierra-Ríos et al., 2020; Harvey and Light, 2015). As outlined by Godbout and Gréhaigne (2021), this dialogue progresses through phases: “Getting it right”, “Judgement”, “Proposal”, “Persuade”, and “Conviction”, encouraging critical thinking and team cohesion.

Guided questioning steers players to focus on key tactical elements, challenges, and action planning (Sierra-Ríos et al., 2020), reinforcing the idea that learning is inherently social and interactive (Sierra-Ríos et al., 2020; Harvey and Light, 2015).

Over time, the GBA framework has evolved into diverse models rooted in active learning, reflection, and meaningful player interaction (Sierra-Ríos et al., 2020; Harvey and Light, 2015). Among these, TGfU remains foundational, linking tactical knowledge, decision-making, and technical execution through purposeful, game-like tasks (Morales-Belando et al., 2022). Its learner-centred foundation has supported its global adoption across varied sporting contexts.

### 1.2 Essential cognitive skills for critical thinking development

The concept of critical thinking has evolved over more than two millennia, becoming a cornerstone in both educational and sporting contexts (Paul and Elder, 2024). Within sport, critical thinking fosters reflective capacities, enabling athletes to make sound and effective decisions in dynamic and often unpredictable environments (Gréhaigne et al., 1999). This foundational skill underpins tactical competence and enhances learning through an ongoing cycle of experience and reflection (Gaviria Alzate et al., 2024a).

Critical thinking integrates both scientific and philosophical dimensions, comprising a set of core cognitive skills that allow individuals to process information effectively and apply it judiciously to guide their behavior (Paul and Elder, 2024). In contrast to rote memorization or passive learning, it demands active analysis, rigorous evaluation, and logical reasoning processes continuously shaped by domain-specific experience (Gaviria Alzate et al., 2024a). The inherent variability of such experience's accounts for individual differences in critical thinking ability.

Elder and Paul (2009) define critical thinking as a disciplined, self-directed process that fosters objectivity through the systematic questioning of assumptions, critical appraisal of diverse information sources, and the formulation of well-reasoned, evidence-based conclusions. Similarly Glaser (1942) views it as a form of reflective reasoning that employs systematic approaches to reach sound, justifiable decisions. Both perspectives highlight the importance of continuous reflection and evidence-based evaluation.

Facione (2020) widely acknowledged framework identifies six core cognitive skills essential to critical thinking: interpretation, analysis, evaluation, inference, explanation, and self-regulation. These interrelated skills underpin effective problem-solving and informed decision-making (see Table 1).

**Table 1:** Essential cognitive skills for critical thinking development.

**Skill**	**Expert consensus**	**Expert Consensus**	**Expert Consensus**
Interpretation	Comprehend and express the meaning or significance of a wide variety of experiences, situations, data, events, judgments, conventions, beliefs, rules, procedures, or criteria.	Categorize decode meaning clarify meaning	What does this mean? How should we understand that (e.g., what was just said or done)? What is the best way to characterize/categorize/classify this? In this context, what was intended by saying/doing that? How can we make sense of this (experience, feeling, or statement)?
Analysis	Identify the intended and actual inferential relationships among statements, questions, concepts, descriptions, or other forms of representation intended to express beliefs, judgments, experiences, reasons, information, or opinions.	Examine ideas identify arguments, identify reasons and claims	Can you restate your reasons for making that claim? What is your conclusion? What are you asserting? Why do you think that? • What are the arguments for and against? What assumptions must we make to accept that conclusion? What is your basis for saying that?
Inference	Identify and secure the elements needed to draw reasonable conclusions; form conjectures and hypotheses; consider relevant information and deduce the consequences flowing from data, statements, principles, evidence, judgments, beliefs, opinions, concepts, descriptions, questions, or other forms of representation.	Query evidence conjectures alternatives, draw logically valid or justified conclusions	Given what we know so far, what conclusions can we draw? Given what we know so far, what can we rule out? What does this evidence imply? If we abandoned/accepted that assumption, how would things change? What additional information do we need to resolve this question? If we believed these things, what would they imply for us in the future? What are the consequences of doing things this way? What are some alternatives we haven't yet explored? let's consider each option and see where it leads Are there any undesirable consequences we can and should foresee?
Evaluation	Assess the credibility of statements or other representations which are accounts or descriptions of a person's perception, experience, situation, judgment, belief, or opinion; and to assess the logical strength of the actual or intended inferential relationships among statements, descriptions, questions, or other forms of representation.	Assess credibility of claims assess the quality of arguments made using inductive or deductive reasoning	How credible is that claim? Why do we believe we can trust what this person says? How strong are those arguments? Do we have our data correct? How confident can we be in our conclusion, given what we know now?
Explanation	State and justify that reasoning in terms of evidential, conceptual, methodological, criteriological, and contextual considerations upon which that results were based; and to present reasoning in the form of cogent arguments.	State results justify procedures present arguments	What were the specific findings/results of the investigation? Tell us how you conducted that analysis How did you arrive at that interpretation? Explain your reasoning to us once again Why do you think (it was the right answer/solution)? How would you explain why this decision was made?
Self-regulation	Self-consciously to monitor one's cognitive activities, the elements used in those activities, and the results educed, particularly by applying skills in analysis and evaluation to one's own inferential judgments with a view toward questioning, confirming, validating, or correcting either one's reasoning or one's results.	Self-examinations self-correction	Our position on this issue is still too vague; can we be more specific? How good was our methodology and how well did we follow it? Is there a way to reconcile these two apparently conflicting conclusions? How good is our evidence? • Okay, before we commit, what are we missing? I'm finding some of our definitions a bit confusing; can we review what we mean by certain things before making any final decisions?

Adapted from Facione (2020).

Educators and coaches play a vital role in cultivating these skills among learners and athletes, directly contributing to greater tactical awareness and understanding (Gréhaigne et al., 1999). Lai (2011) convincingly argues that critical thinking is teachable and can be meaningfully developed through structured and intentional practice. Similarly McBride and Cleland (1998) propose a comprehensive model encompassing broad thinking, causal reasoning, evaluation, planning, and metacognition as central to strategic decision-making in complex environments. Studies by Usra et al. (2023) and Dwyer et al. (2014) confirm that structured game-based learning significantly improves athletes' critical reasoning and reflective analysis skills.

Mahanal et al. (2019) emphasize the critical role of analyzing, evaluating, and restructuring information effectively as a prerequisite for making timely and effective decisions. Evidence consistently shows that targeted training interventions can lead to significant improvements in critical thinking (Mahanal et al., 2019; Sari et al., 2021). Moreover, explicit instructional strategies enhance deeper learning by reducing ambiguity and fostering a clearer understanding of underlying concepts.

The Teaching Games for Understanding (TGfU) model exemplifies an approach that successfully integrates cognitive and social dimensions into the learning process. Usra et al. (2023) reported a 16.14% improvement in critical thinking skills among secondary school students following a TGfU-based intervention. The use of authentic, engaging tasks in such models increases learner motivation, while their involvement in decision-making processes promotes both retention and the development of metacognitive skills (Dwyer et al., 2014).

Importantly, critical thinking in sport transcends technical execution, encompassing higher-order cognitive processes such as strategic and reflective reasoning. These are essential for making informed and adaptive decisions in complex game situations (Mahanal et al., 2019). This aligns with established learning models that emphasize cycles of perception, assimilation, accommodation, and adaptation (Godbout and Gréhaigne, 2021), thereby highlighting the need for active engagement with dynamic environments.

This socio-constructivist stance views learning as an inherently interactive and context-sensitive process in which knowledge is constructed through social interaction and experience. Game-Based Approaches, as previously discussed, promote adaptive learning by encouraging players to co-construct tactical knowledge (Renshaw et al., 2016). Usra et al. (2023) further support this, showing that game-based environments enhance critical thinking by fostering both active engagement and structured reflection. By integrating these principles into their practice, coaches can develop athletes who are not only technically skilled but also critically aware and tactically adaptive, ultimately improving individual and collective performance.

### 1.3 Constructivism and socio-constructivism in sports training

A robust understanding of learning theories is essential for designing effective instructional strategies in sports training (Griffin and Richard, 2023). These theories inform pedagogical decisions, support the adaptation of training approaches, and facilitate the anticipation of learning outcomes.

### 1.4 Constructivism: the individual construction of knowledge

Constructivism posits that individuals actively construct meaning through personal experience and interaction with their environment (Piaget, 1952). Learning is considered an internal process shaped by existing cognitive structures and prior knowledge, with active engagement and reflection as its core components (Gaviria Alzate et al., 2024a; Saleem et al., 2021).

Socratic questioning and critical dialogue are key pedagogical tools within this paradigm (George, 2015). Piaget's stages of cognitive development emphasize the role of interaction in advancing thought, while Bruner underlined the learner's active role in constructing meaning (Saleem et al., 2021). Experimental tasks, such as the three-mountain problem, illustrate the progression from egocentric to decentrated thinking (Piaget and Inhelder, 1956).

### 1.5 Socio-constructivism: the social dimension of learning

Socio-constructivism expands upon constructivist principles by emphasizing the formative role of culture, language, and social interaction in cognitive development (Hickey, 1997; Saleem et al., 2021). Rooted in Vygotsky's sociocultural theory, this view asserts that knowledge is co-constructed through collaborative activity and guided participation within a specific cultural context (Vygotsky et al., 1978).

The concept of the Zone of Proximal Development (ZPD) illustrates how learners can achieve higher levels of understanding with support from more knowledgeable others. Language, in this framework, is a primary tool for mediating learning. Contrary to Piaget's model where cognitive development precedes socialization Vygotsky argued that higher cognitive functions originate in social interaction and are later internalized (Piaget and Inhelder, 1956; Vygotsky et al., 1978).

### 1.6 Socio-constructivism in education and sports training

Socio-constructivism underscores the role of collaboration, dialogue, and shared experience in learning (Hickey, 1997). Educational and sports settings function as communities of practice where knowledge is co-constructed through meaningful interaction (Griffin and Richard, 2023). Within this model, educators and coaches act as facilitators acknowledging learners' prior knowledge, encouraging dialogue, promoting problem-solving, and allowing time for reflection and internalization (Griffin and Richard, 2023). To mitigate power imbalances within communities of practice, TPCT promotes inclusive interaction by rotating speaking turns during reflective discussions, ensuring all voices are heard particularly those from underrepresented groups. This approach aligns with inclusive pedagogical strategies that support equitable cognitive engagement and diverse expression of tactical insight (Lunenburg, 2011; Light and Harvey, 2017).

TPCT redefines the coach as a learning facilitator, encouraging a bidirectional flow of ideas that counters hierarchical dynamics common in elite sport environments (Gaviria Alzate et al., 2024a; Griffin and Richard, 2023). This approach naturally fosters critical thinking, supports dynamic assessment of learners' potential, and promotes inclusive environments responsive to diverse needs. Although time constraints may limit extended reflection in competitive environments, brief, structured discussions embedded in sessions have shown positive cognitive impacts (Light, 2012). Moss et al. (2014) emphasize the mediating role of social interaction and culturally developed tools particularly language in bridging individual cognition and sociocultural context. Griffin and Richard (2023) outline three core principles of socio-constructivist pedagogy:

1. Reality is socially constructed through shared meanings (Vygotsky et al., 1978)
2. Knowledge emerges through collaborative activity.
3. Meaningful learning occurs in authentic social contexts.

### 1.7 The interplay of socio-constructivism and critical thinking in sports

Le and Nguyen (2024) demonstrate the effectiveness of socio-constructivist methods in enhancing critical thinking, with broader implications across disciplines, including sport. Essential features of this approach that support critical thinking include:

✓Interaction and collaboration: peer dialogue fosters perspective-taking and deeper understanding.
✓Application of prior knowledge: new information is assimilated more effectively when linked to existing cognitive structures.
✓Problem-solving: engaging with real tasks enhances analytical reasoning and solution development.
✓Engagement: collaborative settings increase motivation and learner investment.

Lunenburg (2011) affirms the capacity of socio-constructivist strategies to develop higher-order thinking and transfer knowledge to real contexts. In sport, these principles are particularly relevant. Gaviria Alzate et al. (2024a) highlight the role of verbal interaction and co-decision-making in refining athletes' critical thinking. Discussing tactical options, analyzing scenarios collectively, and justifying decisions contribute directly to the development of reflective and strategic thinking.

To summary, constructivism and socio-constructivism offer distinct yet complementary views of learning. While constructivism centers on the individual construction of knowledge, socio-constructivism places emphasis on its social co-construction. When applied to sports training, these perspectives offer a robust foundation for cultivating critical thinking, enhancing problem-solving, and fostering collective learning (Gaviria Alzate et al., 2024a). Environments that promote interaction, build on prior knowledge, integrate authentic challenges, and encourage active participation contribute to the development of more tactically competent and cognitively engaged athletes.

## 2 General description of framework tactical program based on critical thinking (TPCT)

Grounded in socio-constructivist learning theory and critical thinking principles, the Tactical Program Based on Critical Thinking (TPCT) offers a structured yet adaptable framework for training in team sports (Metzler, 2017). Unlike purely conceptual models, TPCT outlines specific, actionable activities designed to cultivate core critical thinking skills—including interpretation, analysis, inference, evaluation, explanation, and self-regulation (Facione, 2020) (see Table 1).

### 2.1 Framework overview

TPCT is underpinned by socio-constructivist principles, emphasizing active learning through collaborative engagement and problem-solving tasks. The program promotes meaningful interactions among players, encouraging shared dialogue around tactical challenges and using reflective questioning techniques to enhance tactical understanding and collective strategy development (Gaviria Alzate et al., 2024a).

TPCT aligns with socio-constructivist principles by facilitating player-led problem-solving in authentic contexts, fostering negotiation of meaning and guided participation, as advocated by Vygotsky (Vygotsky et al., 1978).

### 2.2 The IDEAS model and TPCT implementation

Central to TPCT is (Facione, 2020) IDEAS model, which outlines a five-stage, sequential decision-making process:

✓Identify: recognizing the problem or tactical situation.
✓Determine: gathering relevant data and information.
✓Enumerate: listing potential options.
✓Assess: evaluating the merits and drawbacks of each option.
✓Scrutinize: reflecting on the decision-making process and outcomes.

By integrating the IDEAS model, TPCT systematically promotes structured reflection and collaborative analysis, directly enhancing players' tactical decision-making capabilities (Gaviria Alzate et al., 2024a; Facione, 2020). Table 2 summarizes the step-by-step design of a TPCT training session.

**Table 2:** Step-by-step design of a training session using TPCT.

**Step**	**Description**	**Purpose**	**Method/action**
1. Set Practice Objectives	Begin by introducing and discussing the practice objectives. These should focus on key tactical principles of the game.	Align the group with clear goals and expectations.	Briefly introduce objectives (max 10 minutes), ensuring that the group understands the task. Engage in a group discussion to clarify expectations.
2. Introduce Training Methods	Select appropriate training methods, including small-sided games (SSGs), modified games, and task-based activities.	Engage players in learning through contextualized and task-specific actions.	Explain the different training methods and their goals. For instance, small-sided games focus on decision-making, while modified games target specific skills like passing or positioning.
3. Conduct Small-Sided Games (SSGs)	Implement small-sided games with reduced player numbers, limited space, and specific constraints.	Increase the frequency and quality of tactical decision-making.	Organize games that require players to make rapid decisions under pressure, using constraints like limited touches or positioning rules. Unlike traditional approaches that prescribe fixed tactical responses, TPCT empowers players to generate context-specific strategies through critical reasoning and reflective dialogue (Gaviria Alzate et al., 2024a; Gréhaigne et al., 1999; Gaviria Alzate et al., 2024b)
4. Introduce Modified Games	Use modified games that adjust rules or equipment to emphasize specific tactical behaviors (e.g., passing, positioning).	Develop specific tactical skills through adapted contexts.	Adjust rules (e.g., restrict dribbles to promote positioning) or modify equipment to target specific learning outcomes.
5. Task-Based Activities	Design task-based drills that isolate specific skills or behaviors in a controlled environment.	Bridge the gap between individual skills and team tactics.	Structure drills that allow players to focus on one aspect of the game, such as passing under pressure or defensive positioning.
6. Evaluation of Performance	Set up evaluators to observe and record key actions such as passes, goals, or recoveries.	Track player performance based on measurable outcomes.	Have two evaluators per team to record tactical actions in real-time. These metrics help players understand their performance and areas for improvement.
7. Reflection and Discussion	After each game or activity, facilitate a 3-5 minute reflection period.	Foster critical thinking and group analysis.	Use guiding questions (e.g., “What are we doing well?”) to promote reflection on the game and identify improvements. Encourage players to formulate strategies.
8. Guiding Questions for Reflection	Use structured questions to guide critical thinking and reflection:	Support cognitive development through analysis and problem-solving.	– Are we meeting the objective? (Interpretation) – What are we doing well? (Analysis) – What challenges are we facing? (Inference) – What technical tools do we need? (Evaluation) – What strategy can we propose? (Explanation)
9. Iteration of the Process	Repeat the process for 2-3 more rounds to refine strategies and reinforce learning.	Allow players to apply learned concepts and strategies continuously.	Facilitate a few iterations, allowing players to apply changes to their strategies based on the reflection process. Encourage self-evaluation and peer feedback.
10. Free Play/Unrestricted Game	Conclude the session with a phase of free play to integrate tactical and technical learning.	Enable players to apply learned strategies in a real-game environment.	Provide a context that mirrors actual game conditions, allowing players to experiment with strategies and make independent decisions. Ensure at least 40% of the session time is dedicated to free play.
11. Final Discussion & Debrief	End with a final discussion to review the practice session as a whole.	Reflect on the day's learning and reinforce key tactical principles.	Conduct a brief team debrief, summarizing key lessons learned and discussing areas for future improvement.

Training design and methodologies.

### 2.3 Training design and methodologies

While TPCT has been most frequently applied in football settings with youth players aged 8 to 14 (Gaviria Alzate et al., 2024b, 2025), its underlying principles and methods are designed to be sport-agnostic and adaptable to different competitive levels. The flexibility of its structure enables implementation across a wide range of team sports, including those at both grassroots and elite performance levels.

Each TPCT session begins with a brief introductory briefing (under 10 min) that outlines specific objectives in line with long-term talent development principles (Ericsson and Pool, 2016). The core methodologies include:

✓Small-sided games (SSGs): these formats with reduced space and fewer players intensify decision-making demands and enhance tactical awareness (Hill-Haas et al., 2011).
✓Modified games: adaptations to standard rules or equipment target specific learning outcomes and reinforce tactical concepts (Davids et al., 2013).
✓Task-based activities: focused drills bridge individual skill development and overall team cohesion by isolating and honing technical and tactical behaviors (Renshaw et al., 2016).

To accommodate diverse skill levels, TPCT applies adjustable constraints in training tasks and promotes guided reflection tailored to each player's developmental needs (Davids et al., 2013; O'Connor et al., 2017). Training sessions are designed to be adaptive. Coaches adjust activities to meet the group's specific needs, ensuring optimal engagement through evidence-based coaching practices (Davids et al., 2013). The duration of SSGs is pre-calibrated, with pilot sessions refining time requirements for maximum effectiveness. Objective evaluation is achieved by using designated evaluators rotated among players to track key performance indicators such as completed passes, goals, and ball recoveries (O'Connor et al., 2017; Côté et al., 2007).

### 2.4 Reflection and tactical adaptation

Immediately following each SSG, a structured discussion (lasting 3–5 min) is conducted. This debrief allows both the team and individual players to evaluate performance and collaboratively refine tactical strategies. Although coaches initially guide these discussions, the aim is to progress toward player-led reflections, thereby promoting ownership of learning (Light, 2012). Guiding questions scaffold critical thinking during these sessions by addressing:

✓Whether the intended objective is achieved (Interpretation/Identification).
✓Which performance aspects were successful (Analysis/Determination).
✓Key current challenges (Inference/Enumeration).
✓Technical skills needing refinement (Evaluation).
✓Strategic adjustments to optimize success (Explanation/Examination).

Quantitative feedback from evaluators, grounded in measurable performance data, further informs these reflective discussions (O'Connor et al., 2017). Multiple iterations of similar training scenarios ensure that refined strategies lead to demonstrable improvements in key performance variables, reinforcing socio-constructivist learning through practical application (Renshaw et al., 2016; Light, 2012).

### 2.5 Free play phase

Each training session concludes with a free play phase comprising at least 40% of the total session time to provide an opportunity for players to integrate learned tactical adaptations within realistic, game-like contexts (Davids et al., 2013). This balance between structured learning and adaptive free play is crucial for optimizing both decision-making capabilities and long-term performance development (Ford et al., 2010).

## 3 Final considerations, conclusions, and practical implications

TPCT presents an innovative approach to enhancing tactical performance by deliberately cultivating critical thinking skills. By engaging players in reflective, contextually rich learning experiences, TPCT supports more effective decision-making under pressure. Its player-centered, socio-constructivist framework positions coaches as facilitators empowering athletes to collaboratively generate and refine strategies, which in turn enhances adaptability and tactical creativity on the field (Chow et al., 2015; Williams and Hodges, 2005).

Empirical evidence has substantiated the effectiveness of TPCT in youth sport contexts. A case study conducted with under-14 football players (Gaviria Alzate et al., 2024b) demonstrated notable gains in tactical efficiency and sport-specific knowledge, while a subsequent quasi-experimental study with under-8 athletes (Gaviria Alzate et al., 2025) identified improvements in ball control and passing accuracy. Furthermore, preliminary findings from an ongoing doctoral project involving elite underwater rugby athletes have shown significant positive changes in tactical efficiency indices and critical thinking skills. These collective results reinforce the programme's relevance across age groups and performance levels, validating its application as both a pedagogical and performance-enhancing intervention.

Despite its structured design and flexible application, TPCT implementation may be constrained by contextual factors. Coaches may face challenges related to limited time for post-task reflection, varying degrees of familiarity with socio-constructivist pedagogy, and institutional constraints such as facility access or curricular rigidity. To overcome these limitations, TPCT recommends incorporating concise, targeted reflection periods and adapting implementation progressively according to the coach's experience and the training environment.

Unlike traditional Game-Based Approaches such as Teaching Games for Understanding (TGfU), which create favorable conditions for reflection and decision-making, TPCT deliberately targets the structured development of the core cognitive skills that underpin critical thinking. These include interpretation, analysis, inference, evaluation, explanation, and self-regulation. By embedding the IDEAS model within training dynamics and facilitating structured dialogue, TPCT moves beyond promoting tactical awareness to systematically enhancing athletes' metacognitive and reasoning capacities. This positions TPCT not simply as a pedagogical strategy, but as a cognitive-educational framework designed to build the very foundation of intelligent gameplay.

In contrast to traditional, decontextualized training methods (Pol et al., 2020), TPCT's focus on co-constructing tactical strategies within realistic game situations aligns with the findings of Ribeiro et al. (2019) regarding the importance of team synergy. While sharing similarities with Teaching Games for Understanding (TGfU) (Renshaw et al., 2016; Abad Robles et al., 2020; Ortiz et al., 2023), TPCT uniquely prioritizes the explicit cultivation of critical thinking skills through structured reflection and game-based tasks.

Structured reflection is fundamental for developing robust critical thinking (Quarmby and Luguetti, 2023). By bridging academic theory and practical coaching, TPCT empowers players to analyze complex game scenarios, critically evaluate options, and collaboratively develop effective tactical solutions (Dwyer et al., 2014).

### 3.1 Practical implications include:

✓Program application: TPCT offers a structured framework that integrates cognitive and tactical skills for lasting learning outcomes.
✓Constructivist integration: by incorporating game-related constraints and fostering reflective discussions, TPCT enhances tactical awareness and adaptive learning.
✓Tactical specificity: the program's design is adaptable to specific sports demands, enabling coaches to target and reinforce key tactical principles.

Ultimately, TPCT strengthens the interplay between players and their environment, optimizing both cognitive and tactical performance by promoting strategic proposal generation and task-conditioned responses (Arias et al., 2016; Davids et al., 2021). This framework offers coaches a valuable, innovative approach for enhancing team performance through reflective practice and collaborative learning (Vygotsky et al., 1978; Light and Harvey, 2017; Jones et al., 2016).
